# Blocking TRPA1 in Respiratory Disorders: Does It Hold a Promise?

**DOI:** 10.3390/ph9040070

**Published:** 2016-11-05

**Authors:** Indranil Mukhopadhyay, Abhay Kulkarni, Neelima Khairatkar-Joshi

**Affiliations:** Biological Research, Glenmark Research Centre, Glenmark Pharmaceuticals Ltd. Navi Mumbai, Maharashtra 400709, India; Indranil.Mukhopadhyay@glenmarkpharma.com (I.M.); Abhay.Kulkarni@glenmarkpharma.com (A.K.)

**Keywords:** TRPA1, airway inflammation, airway sensory responses, asthma, COPD, cough

## Abstract

Transient Receptor Potential Ankyrin 1 (TRPA1) ion channel is expressed abundantly on the C fibers that innervate almost entire respiratory tract starting from oral cavity and oropharynx, conducting airways in the trachea, bronchi, terminal bronchioles, respiratory bronchioles and upto alveolar ducts and alveoli. Functional presence of TRPA1 on non-neuronal cells got recognized recently. TRPA1 plays a well-recognized role of “chemosensor”, detecting presence of exogenous irritants and endogenous pro-inflammatory mediators that are implicated in airway inflammation and sensory symptoms like chronic cough, asthma, chronic obstructive pulmonary disease (COPD), allergic rhinitis and cystic fibrosis. TRPA1 can remain activated chronically due to elevated levels and continued presence of such endogenous ligands and pro-inflammatory mediators. Several selective TRPA1 antagonists have been tested in animal models of respiratory disease and their performance is very promising. Although there is no TRPA1 antagonist in advanced clinical trials or approved on market yet to treat respiratory diseases, however, limited but promising evidences available so far indicate likelihood that targeting TRPA1 may present a new therapy in treatment of respiratory diseases in near future. This review will focus on in vitro, animal and human evidences that strengthen the proposed role of TRPA1 in modulation of specific airway sensory responses and also on preclinical and clinical progress of selected TRPA1 antagonists.

## 1. TRPA1 Receptor

TRPA1 ion channel and its biology is known for more than a decade now. It has attracted academic scientists due to its interesting tissue distribution and functions. TRPA1 is abundantly expressed in peripheral nervous system and has demonstrated a chemosensory function. Hence, it has been assigned a role of “warning system” against external as well as internal assaults. Industrial researchers are interested in TRPA1 as a target for therapeutic intervention due to its implicated role in miscellaneous diseases. Evidence for potential role of TRPA1 in pain signaling based on TRPA1 knockout (TRPA1 KO) mice and siRNA studies emerged much earlier compared to its involvement in airway diseases—which is relatively recent and still emerging. TRPA1 is highly expressed on pulmonary innervation—an anatomically relevant region for respiratory diseases, and could be one of the major players in orchestration of airway inflammatory response. A large number of recent studies have implicated TRPA1 in the pathogenesis of several respiratory diseases including chronic cough, asthma, COPD, allergic rhinitis and cystic fibrosis. Blockade of TRPA1 is perceived as a novel strategy for its therapeutic intervention.

## 2. TRPA1—Expression and Activation in Airways

TRPA1 was first identified in cultured human lung fibroblasts [1]. Later on, TRPA1 expression on mouse lung was reported, albeit to a lesser extent compared to other tissues [2]. TRPA1 is abundantly expressed on the innervations of almost entire respiratory tract including the C-fibers of the trigeminal and vagal ganglia, which innervate upper regions of oral cavity and oropharynx and on conducting airways in the trachea, bronchi, terminal bronchioles, respiratory bronchioles, alveolar ducts and alveoli. C-fibers largely “sense” the presence of potentially toxic inhaled irritants and toxicants. TRPA1 was initially recognized as an irritant sensing ion channel on majority of vagal nociceptive C-fibers in the bronchopulmonary region [3]. It is well known now that unlike traditional chemoreceptors, TRPA1 is activated by a wide variety of structurally different molecules with high chemical reactivity spanning from exogenous irritants to endogenously produced reactive reagents [4,5,6,7,8,9,10,11]. Functional presence of TRPA1 on non-neuronal cells got recognized recently. TRPA1 is shown to be expressed on lung fibroblasts, epithelial and smooth muscle cells [12,13,14,15].

The role of TRPA1 in airway pathologies has been corroborated by studies using the TRPA1 KO mice and TRPA1 antagonists. In wild-type mice, airway exposure to hypochlorite or Hydrogen peroxide (H_2_O_2_) evoke respiratory depression as manifested by a reduction in breathing frequency and increase in end expiratory pause, both of which were attenuated in TRPA1 KO mice [6]. A host of chemicals are known to be specifically associated with respiratory diseases and airway reflex responses such as coughing, sneezing and bronchoconstriction. Acrolein and crotonaldehyde induce strong tussive response in animals as well as in man. H_2_O_2_, 4-Hydroxynonenal (4-HNE), photochemical smog, zinc, toluene diisocyanate are associated with allergic asthma in humans. All the above listed chemicals are direct and potent activators of TRPA1. α,β-unsaturated aldehydes are earliest recognized TRPA1 activators, and are detected in air spaces, breath, sputum, lungs, and blood from patients with asthma as well as COPD. Fragrance chemicals like menthol, 1,8-Cineole, borneol, fenchyl alcohol etc. are associated with TRPA1 activation and play a key role in allergic reactions [16]. Cigarette smoke, wood smoke, diesel exhaust, and other combustion-derived particles activate TRPA1, causing irritation and inflammation in the respiratory tract [17,18]. TRPA1 is a molecular sensor for wood smoke particulate and several chemical constituents such as 3,5-ditert-butylphenol, coniferaldehyde, formaldehyde, perinaphthenone, agathic acid, and isocupressic acid are TRPA1 agonists [18]. Nassini R et al. [14] demonstrated that TRPA1 is expressed in non-neuronal cells in human and murine airways, and promote a non-neurogenic inflammatory response. This raises an alternate possibility that airway inflammation may also be promoted by non-neuronal mediators. Thus, TRPA1 ion channel with its wide range of expression in neuronal and non-neuronal cells and its activation by several exogenous and endogenous stimuli relevant to airway sensory responses may be a major regulator in driving several respiratory diseases.

## 3. TRPA1 in Chronic Cough

**Figure 1:**
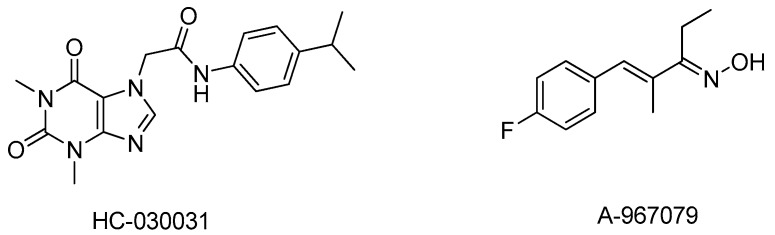
Chemical structure of Hydra Biosciences (HC-030031) and Abbott (A-967079).

Cough is a vagally mediated defensive mechanism to protect the airway to clear respiratory tract from continuously exposed airborne environmental irritants. Despite wide prevalence of chronic cough, therapy options are very inadequate and often symptomatic. Currently available treatments such as dextromethorphan, hydrocodone and codeine are inadequate due to limited efficacy, central nervous system (CNS) side effects or abuse liability, respiratory depression and gastrointestinal disturbances. The paucity, if not absence of effective medicine to treat cough is mainly because the molecular mechanisms that orchestrate chronic cough are not clearly understood in experimental animal models as well as in man. Scientists are attempting to shed some light on the possible mechanism behind the occurrence of chronic cough. A number of TRP channels (TRPA1, TRPV1 and TRPV4) have been linked to sensory perception relevant to cough response [19]. A large number of in vitro and in vivo studies have recently indicated important role of TRPA1 activation in driving cough reflex. Acrolein and crotonaldehyde are contained in cigarette smoke and polluted air which are well recognized cough inducers. TRPA1 agonists are shown to induce activation of recombinant human TRPA1 channels in vitro in human embryonic kidney cells (HEK293), depolarization of murine, guinea pig and human vagal sensory nerves and produce cough in healthy human volunteers [20]. Allyl isothiocyanate (AITC), acrolein, crotonaldehyde and cinnamaldehyde—all are potent TRPA1 agonists and shown to induce dose dependent and robust tussive response in guinea pigs which was attenuated by non-selective TRP channel blockers (camphor, gentamicin) as well as by selective TRPA1 antagonist from Hydra Biosciences—HC-030031 (Figure 1) [9]. The cough response however remained unchanged upon administration of capsazepine which is a TRPV1 antagonist as well as desensitizing TRPA1 agonist [21]. We demonstrated direct in vitro activation of TRPA1 receptor by citric acid which is routinely used to evoke cough in preclinical and clinical studies [22]. Citric acid induced tussive response in guinea pigs was inhibited by a potent and selective TRPA1 antagonist from our laboratory, GRC 17536 (structure not disclosed). Anti-tussive effect of other TRPA1 antagonists is also demonstrated in animal cough model. Recently, a poster from Almirall at European Respiratory Society Conference (2015) reported a potent oral TRPA1 antagonist-Compound A, that showed inhibition of isolated guinea pig vagus nerve depolarization with EC_50_ of 80 nM. Furthermore, in the in vivo model of AITC induced cough, Compound-A showed dose dependent inhibition of cough response with ED_50_ of 0.17 mg/kg dose [23].

Importantly, TRPA1 mediated tussive response is associated with both exogenous TRPA1 agonists as well as endogenous biochemicals that are produced during diseases associated with cough. For example, prostaglandin E2 (PGE_2_) and bradykinin are produced during tissue inflammation. Mucoid secretions containing similar pro-tussive inflammatory mediators are produced in patients with post nasal drip syndrome (PNDS). 4-HNE, reactive oxygen species (ROS), reactive nitrogen species (RNS), 15-deoxy-delta-12,14-prostaglandin J2 (15d-PGJ_2_) are produced in the airways during asthma, COPD and in the esophagus during gastroesophageal reflux disease (GERD) [24] and lastly, toll like receptors (TLRs) that are produced during post viral cough condition. [25,26,27]. Thus, TRPA1 could have a central role in producing chronic cough associated with diverse pathologies but via a common mechanism of vagal nerve activation due to its activators prevailing during specific diseases as described above [9,20,28,29]. Hence TRPA1 has emerged as one of the most promising targets for the development of medicines to treat chronic cough.

## 4. TRPA1 and Asthma

Asthma is an inflammatory disease of the lungs characterized by bronchoconstriction and airway hyperreactivity, leading to shortness of breath, wheezing, and coughing. It is triggered by exposure to allergen or irritant and manifested as episodes or attacks of airway narrowing that are mediated by airway nerves and muscles. Studies focusing on airway smooth muscle and epithelial function strongly indicate involvement of immunogenic and neurogenic mechanisms in airway inflammation. TRPA1 could be a key integrator of immune and neuronal signaling in the airways [30].

Various noxious chemicals and environmental/industrial irritants that activate TRPA1 happen to be triggers for asthma or reactive airways dysfunction syndrome (RADS) and are known to worsen asthma attacks [31]. Toluene diisocyanate is a potent TRPA1 activator and exposure to this chemical is the leading cause of occupational asthma. Exposure to cigarette smoke strongly correlates with asthma severity. Cigarette smoke ingredients-acrolein and crotonaldehyde activate native and recombinant TRPA1 and produce tracheal extravasation in wild type animals in TRPA1 dependent manner. Asthmatics are sensitive to photochemical smog that contains acrolein and other oxidant species which are also known to activate TRPA1. Several TRPA1 activators or sensitizers are endogenously produced in lungs of patients which are also efficient triggers of asthma e.g., H_2_O_2_, 4-HNE, cyclic prostaglandin PGJ_2_ and bradykinin. Elevated levels of bradykinin and nerve growth factor (NGF) are found in bronchoalveolar lavage of asthma and rhinitis patients while elevated 4-HNE and acrolein are detected in lungs, air spaces, breath, sputum and blood of asthma and COPD patients. Experiments with TRPA1 KO mice revealed reduced airway infiltration by inflammatory leukocytes accompanied by decreased production of pro-inflammatory cytokines and neuropeptide release in the airways in the ovalbumin (OVA) asthma model [30]. In the wild type mice that developed asthma upon OVA challenge, pharmacological blockade of TRPA1 receptor with HC-030031 reiterated the above observation. HC-030031 also inhibited OVA-induced late asthmatic response in dose dependent manner in B/N rats [32]. During late asthmatic response, allergen challenge is implicated to trigger airway sensory nerves via TRPA1 activation to further initiate a central reflex event. A couple of recent posters presented in conferences have reported similar findings as above using TRPA1 selective antagonists [33,34]. GRC 17536 showed significant inhibition of lung eosinophilia, mucus production and airway hyperresponsiveness in mouse asthma model, and, inhibition of eosinophils and early airway reactivity in guinea pig allergic asthma model [33]. Another TRPA1 antagonist from Cubist Pharmaceuticals, CB-625 (structure not disclosed), was effective in reducing the late asthmatic response and antigen induced airway hyperresponsiveness upon oral dosing in sheep model of asthma [34]. Epidemiological evidence has linked therapeutic acetaminophen doses with risk of COPD and asthma [35]. Recent studies by Nassini et al. [36] demonstrated that *N*-acetyl-p-benzoquinoneimine (NAPQ1), a metabolite of acetaminophen, evoked pro-inflammatory responses in the lung. They further demonstrated that NAPQ1 selectively activates TRPA1, and selective inhibition of TRPA1 abated the pro-inflammatory effect of acetaminophen. It remains to be seen if TRPA1 antagonists attenuate asthma in clinics.

## 5. Role of TRPA1 in COPD

COPD is a disease characterized by increased infiltration of inflammatory cells in the lung, increased oxidative and nitrosative burden in the lung and parenchymal destruction, associated with progressive decline in lung function. COPD is strongly associated with smoking status of an individual but other environmental factors such as bio mass fuel exposure are also implicated in the etiology of COPD [37].

TRPA1 is shown to be a major neuronal sensor for oxidants in the airways [6]. Various pollutants, oxidants, cigarette smoke extract (CSE) and cigarette smoke (CS) ingredients such as acrolein, crotonaldehyde, 4-HNE that are associated with the pathobiology of COPD, are potent agonists of TRPA1. Some of these are associated with interleukin 8 (IL8) release in the human macrophagic cell lines [7,38]. Our TRPA1 antagonist GRC 17536 was effective in inhibiting CSE, crotonaldehyde and acrolein induced Ca^2+^ influx in vitro and AITC induced Ca^2+^ influx and IL8 production in human lung fibroblast cells (CCD19-Lu) and human pulmonary alveolar epithelial cell line (A549) [22]. CSE induced increase in TRPA1 expression and IL8 release in human bronchial epithelial cells (HBEC) was attenuated by HC-030031 [39].

Further corroborating evidence to support the role of TRPA1 in COPD came from the studies in animal models of COPD. CS and acrolein induced release of keratinocyte chemoattractant (KC) (CXCL1/GRO alpha; mouse homolog of human IL-8) in bronchoalveolar lavage fluid (BALf) of mice was significantly reduced in TRPA1 KO mice [14]. Pretreatment of animals with HC-030031 showed protection from CS induced plasma protein extravasation whereas capsazepine was not effective suggesting selective involvement of TRPA1 in inducing CS associated inflammation. When C57BL/6 mice was exposed to CS for 4 weeks, significant increase in TRPA1 mRNA was observed in trigeminal and nodose/jugular ganglia of CS-exposed mice with a positive correlation to cellular infiltration. An increase in leukocytes, macrophages and neutrophils was observed in BALf of mice [40]. Increased immunostaining for TRPA1 was observed in wild type mice chronically exposed to CS whereas TRPA1 KO mice revealed reduced inflammation and structural changes [39]. All these observations suggest strong involvement of TRPA1 in mediating CS induced inflammation in the animal models.

Although COPD is directly linked to the smoking history, recently it was shown that other factors like biomass fuels and biomass smoke exposure is also a risk factor for COPD development [41]. Wood smoke and its chemical constituents are activators of TRPA1 and are known for their damaging effects on respiratory system. Wood smoke particulate matter (WSPM) at low concentration, which is relevant in terms of potential human exposure levels are known to activate TRPA1 [18]. There is ample evidence to show involvement of TRPA1 in COPD and TRPA1 antagonists seem promising treatment options in clinical management of COPD.

## 6. Role of TRPA1 in Allergic Rhinitis

Allergic rhinitis and asthma are chronic inflammatory atopic disorders characterized by overlapping symptoms and pathophysiology. Toluene diisocyanate (TDI) is a highly reactive chemical associated with respiratory symptoms resembling allergic rhinitis and a most prevalent cause of occupational asthma [42,43]. Exposure of mice to TDI showed decreased breathing rates, increased cytokines accompanied by increased eosinophils and allergic inflammation in nasal cavity [44]. It is an effective TRPA1 activator with in vitro EC_50_ of 10 µM which is a physiologically relevant concentration known to cause decreased breathing rate and sensory irritation in mice. Interestingly, these symptoms were absent in TRPA1 KO mice [45] suggesting an important role of TRPA1 in allergic or occupational asthma and rhinitis. A recent study showed increased level of TRPA1 mRNA in subjects with rhinitis compared to normal controls (*p* = 0.03) suggesting potential involvement of TRPA1 in allergic rhinitis [46].

## 7. Role of TRPA1 in Cystic Fibrosis

Cystic fibrosis (CF) is a disease associated with mutation in the gene for cystic fibrosis transmembrane conductance regulator (CFTR) and can affect many organs. When upper and lower airways are involved, it causes impaired mucociliary clearance leading to mucus plugging in the airways, failure in effective clearing of inhaled bacteria and lung neutrophilia. Respiratory failure is a major cause of mortality associated with CF. Recently TRPA1 expression was reported on different epithelial cells obtained from lung tissues of CF patients [47]. IL8 expression was observed in the bronchial epithelial cells co-expressing TRPA1. Furthermore TRPA1 specific inhibitors from Hydra Biosciences (HC-030031) and Abbott (A-967079) (Figure 1) [48] inhibited *P. aeruginosa* induced transcription of IL8, IL1b, IL6 and tumor necrosis factor α (TNFα) in A549 and human cystic fibrosis cell line (CuFi-1). *P. aeruginosa* is a gram negative bacteria and releases lipopolysaccharide (LPS) which is a direct TRPA1 agonist and sensitizer [49]. Silencing TRPA1 significantly reduced the release of IL-8, IL-1β and TNF-α from HBECs from CF patients. This is the first study which revealed the potential of TRPA1 antagonists in controlling inflammation associated with CF.

## 8. TRPA1 Antagonists: What’s the Status?

Till date, there is limited published literature showing efficacy of TRPA1 antagonists in respiratory diseases. The accumulating evidence supporting TRPA1 as an attractive target in respiratory disease has resulted in large number of small molecules patents (Table 1). Some of the compounds have been evaluated in animal models of respiratory diseases as summarized in earlier sections and the data is very encouraging. However it remains to be seen if such promising results in animal will eventually translate to efficacy in humans. Until then, translational studies to assess their pharmacological effects on patient derived lung tissues ex vivo could strengthen the claim of their potential utility to treat human respiratory diseases. Such study tools are established for assessing translatability of TRPA1 antagonist pharmacology in pain where in calcitonin gene-related peptide (CGRP) release from human dental pulp is assayed as a biomarker. Recently, TRPA1 antagonist—GRC 17536 from our laboratory has shown positive proof of concept in reducing peripheral diabetic neuropathic pain in patients with intact nerves [50]. The clinical features of chronic pain and refractory chronic cough seem overlapping. Clinical presentation of chronic pain typically includes paraesthesia (abnormal sensation in the absence of a stimulus), hyperalgesia (pain triggered by a low exposure to a known painful stimulus), and allodynia (pain triggered by a non-painful stimulus) and shows similarities with the clinical features of refractory chronic cough, such as an abnormal throat sensation or tickle (laryngeal paraesthesia), increased cough sensitivity in response to known tussigens (hypertussia), and cough triggered in response to non-tussive stimuli such as talking or cold air (allotussia). Gabapentin, which is used to treat neuropathic pain, is also reported to show antitussive effect in human chronic cough patients recently [51]. Hence there is optimism to believe that TRPA1 antagonists could work in treatment of chronic cough.

As far as COPD is concerned, cigarette smoke and wood smoke have a close and proven involvement in COPD pathobiology. Constituents of cigarette smoke and wood smoke are potent activators of TRPA1. Hence, TRPA1 antagonists such as GRC 17536, which have started to show promising clinical benefits in human pain proof of concept trials, could also be effective in treating respiratory diseases. Several pharmaceutical companies have active drug discovery programs targeting TRPA1 receptor for potential treatment of miscellaneous respiratory disorders (Table 1).

## 9. TRPA1 Antagonists: Any Safety Concerns?

TRPA1 KO mice are viable, normal in appearance and fertile with no auditory dysfunction [4,52]. Unlike TRPV1 or TRPM8, TRPA1 is not involved in body temperature regulation at basal level or under cold challenge [53,54]. Hence TRPA1 antagonists are expected to be devoid of adverse effects on body temperature unlike the TRPV1 antagonists (hyperthermia) or TRPM8 antagonists (hypothermia). Human healthy volunteers administered with CB-625 show normal body temperature and also unimpaired thermosensation in heat and cold tests [55]. This is in line with TRPA1 KO mice data that exhibits unimpaired heat and cold sensitivity [4]. Also, wild type (WT) animals in spinal nerve ligation (SNL) nerve injury model when treated with antisense TRPA1 were protected against cold hyperalgesia while showing no effect on normal cold sensitivity [56]. Animals treated with TRPA1 antagonist have normal performance in hot and cold plate tests and no paradoxical sensation [53].

Although TRPA1 is predominantly expressed in sensory neurons, it is also expressed in other non-neuronal tissues, albeit at a much lower level. This raises a question if targeting TRPA1 could have unintended/unrecognized adverse effects on physiological functions or on known protective functions such as sensing environmental irritants. TRPA1 KO mice are recently reported to show physical hyperactivity [57]. Since several TRPA1 antagonists are at different stages of evaluation including chemical synthesis, preclinical studies and clinical trials, their long term safety in patients is likely to be addressed soon.

## 10. Summary

**Figure 2:**
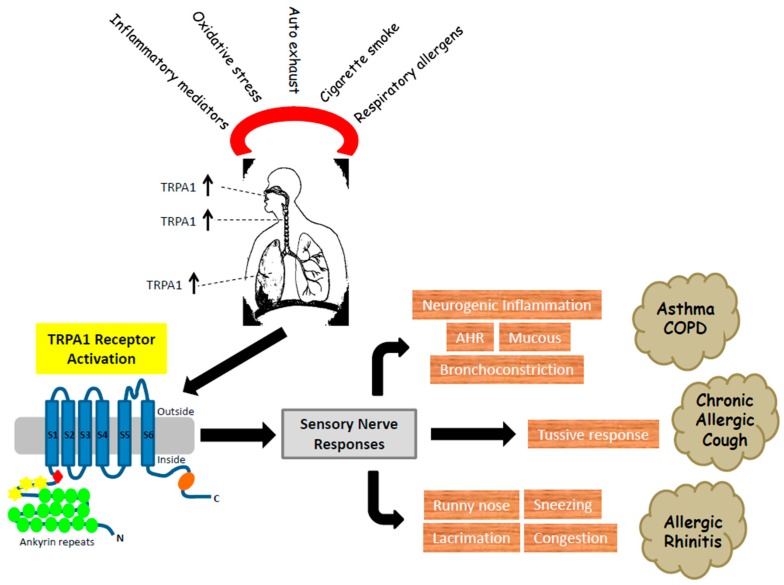
TRPA1 in respiratory diseases.

The role of TRPA1 in the respiratory system has been summarized in Figure 2. TRPA1 is expressed on pulmonary innervation - an anatomically relevant region for respiratory diseases and implicated in the orchestration of inflammatory response in animal models of airway diseases, including chronic cough, asthma, COPD, allergic rhinitis and cystic fibrosis. TRPA1 can remain activated chronically due to elevated levels and continued presence of endogenous ligands and pro-inflammatory mediators. Phenotype of TRPA1 KO mice in allergen-induced asthma model and CS model is in line with this since these animals show reduced leukocyte infiltration in the airways, reduction of cytokine and mucus production.

Preclinical performance of TRPA1 antagonists in respiratory disease models is promising. There is no TRPA1 antagonist in advanced clinical trials or approved on market yet to treat respiratory diseases. We are in the wait to see if they can attenuate undesirable airway sensory responses in man and additionally if they are capable of reversing disease progression and outperform current standard of care such as anti-inflammatory drugs. Available evidences and underlying science indicate likelihood that they may live up to the expectations and help clinicians in treatment of chronic airway diseases with unmet medical need in near future.

## Figures and Tables

**Table 1:** TRPA1 in Respiratory Diseases: A Patent Update.

Patent Number	Owner Companies	Indications
WO2013084153; WO2013014597; WO2012176143; WO2012172475; WO2012176105; WO2011132017; WO2014203210; WO2010125469; WO2010004390; WO2010109287; WO2013183035; WO2009118596; WO2012085662; WO2009144548; WO2011114184	Glenmark Pharmaceuticals SA	Asthma; COPD; Bronchitis, COPD, Cough, Respiratory disorder
WO2009140517; WO2010132838; WO2007073505; WO2010039289; WO2015164643; WO2016044792; WO2010036821	Hydra Biosciences Inc.	Asthma, Cough; Respiratory disease; Asthma; COPD; Lung injury
WO2009071631; WO2010141805	Janssen Pharmaceutica NV	COPD; Lung disease; Cough
WO2015052264; WO2014060341; WO2014056958; WO2014049047; WO2014072325	F Hoffmann-La Roche AG; Hoffmann-La Roche Inc; Roche Holding AG Genentech Inc.	Asthma; COPD; Cough; Allergic rhinitis; Respiratory disease; Bronchospasm
WO2015144976; WO2012152983; WO2014053694; WO2015144977	Orion Corp	Asthma; COPD; Cough
WO2013023102; WO2014113671	Cubist Pharmaceuticals Inc.; Hydra Biosciences Inc.	Asthma; COPD; Respiratory disease
WO2015115507; WO2014098098; WO2013108857	Ajinomoto Co. Inc.	Asthma; COPD; Cough; Lung disease
JP2014024810	Kao Corp	Respiratory failure
WO2009089082	AbbVie Deutschland GmbH & Co KG; Abbott Laboratories	Lung disease
WO2016028325	Duke University; University of California	Fibrosis
WO2015155306	Almirall Prodesfarma SA	Respiratory disease
WO2014135617	Ario Pharma Ltd; PharmEste SRL	Asthma; COPD; Cough

## Abbreviations

TRPA1	Transient Receptor Potential Ankyrin 1
COPD	Chronic obstructive pulmonary disease
TRPA1 KO	TRPA1 knockout
H_2_O_2_	Hydrogen peroxide
4-HNE	4-Hydroxynonenal
CNS	Central nervous system
HEK293	Human embryonic kidney cells
AITC	Allyl isothiocyanate
PGE_2_	Prostaglandin E2
PNDS	Post nasal drip syndrome
ROS	Reactive oxygen species
RNS	Reactive nitrogen species
15d-PGJ_2_	15-deoxy-delta-12,14-prostaglandin J2
GERD	Gastroesophageal reflux disease
TLRs	Toll like receptors
RADS	Reactive airways dysfunction syndrome
NGF	Nerve growth factor
OVA	Ovalbumin
NAPQ1	*N*-acetyl-p-benzoquinoneimine
CSE	Cigarette smoke extract
CS	Cigarette smoke
IL8	Interleukin 8
CCD19-Lu	Human lung fibroblast cells
A549	Human pulmonary alveolar epithelial cell line
HBEC	Human bronchial epithelial cells
KC	Keratinocyte chemoattractant
BALf	Bronchoalveolar lavage fluid
WSPM	Wood smoke particulate matter
TDI	Toluene diisocyanate
CF	Cystic fibrosis
CFTR	Cystic fibrosis transmembrane conductance regulator
TNFα	Tumor necrosis factor α
CuFi-1	Human cystic fibrosis cell line
LPS	Lipopolysaccharide
CGRP	Calcitonin gene-related peptide
WT	Wild type
SNL	Spinal nerve ligation
